# Rapidly evolving cerebral edema and hyperperfusion in a patient with dural arteriovenous fistula

**DOI:** 10.1007/s13760-022-01952-x

**Published:** 2022-04-24

**Authors:** Eva Bürkle, Tobias Lindig, Ulrike Ernemann, Tim W. Rattay

**Affiliations:** 1 Department of Diagnostic and Interventional Neuroradiology, University Hospital Tübingen, Hoppe-Seyler-Str. 3, 72070 Tübingen, Germany; 2 Department of Neurodegenerative Disease, Hertie-Institute for Clinical Brain Research, and Center for Neurology, University of Tübingen, Tübingen, Germany; 3 German Center of Neurodegenerative Diseases (DZNE), Tübingen, Germany

## Introduction

Dural arteriovenous fistulas are usually acquired, abnormal arteriovenous shunts within the dural leaflets. Their clinical manifestation depends on the localization and the venous drainage pathway [1]. Their risk of hemorrhage increases with presence of retrograde cortical drainage [2].

We report the case of a 69-year-old man, who was brought to us with a seizure. The first computed tomography (CT) was not conclusive. The follow-up imaging with magnetic resonance imaging (MRI) 5, respectively, 6 days later showed a rapidly increasing edema within the occipital lobe (excluding the cortex) and enabled us to make the diagnosis of a dural arteriovenous fistula, which was consecutively verified by digital subtraction angiography (DSA) followed by embolization.

## Case presentation

**Fig. 1:**
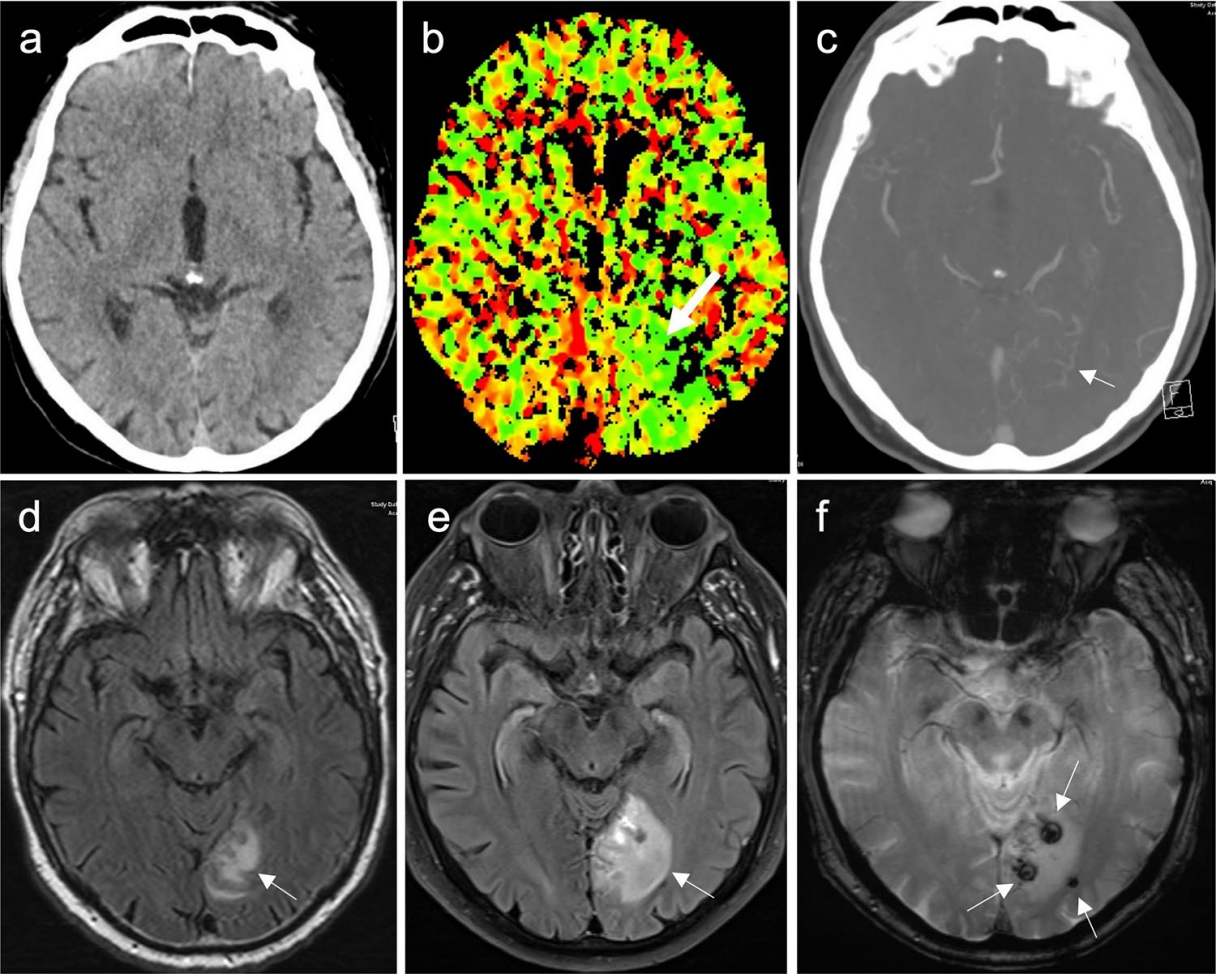
**a–c** Axial views of the first CT: **a** native CT, soft tissue window; **b** corresponding CTP; **c** corresponding CTA, which shows no hemorrhage or edema occipital. The CTP shows an hyperperfusion of the left occipital lobe, the CTA reveals one tortuous vessel in the described area (all specified findings are marked by an arrow in the corresponding picture). **d–f** Follow-up-MRIs (five and six days after the first CT): **d** axial FLAIR with T2-hyperintense edema (arrow); **e** follow-up axial FLAIR MR image one day after **d**, with an increasing volume of the edema; **f** hemosiderin depositions indicate hemorrhages

**Fig. 2:**
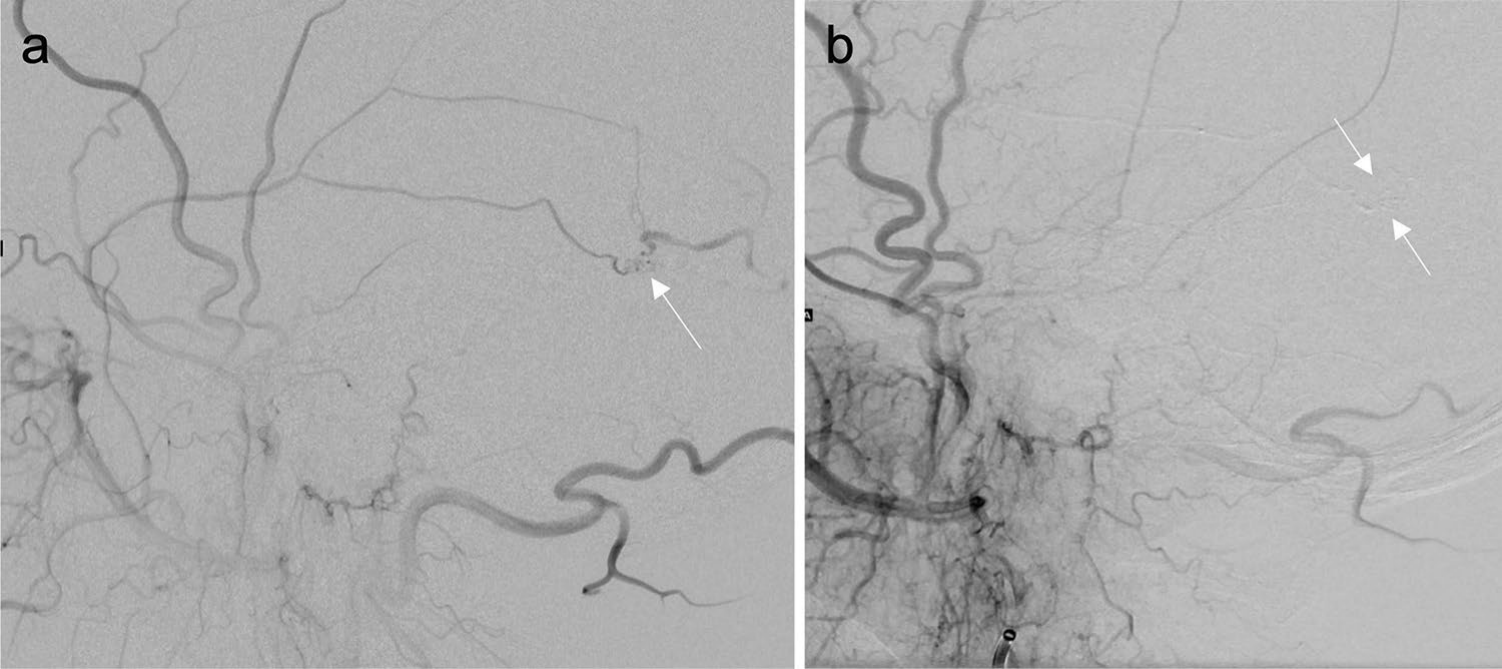
**a**, **b** Cerebral DSA in lateral views: **a** DSA of the middle meningeal artery with two small branches leading to the arteriovenous fistula (arrow). **b** DSA after embolization of the fistula (arrows)

A 69-year-old man was brought to our emergency department by ambulance with decreased vigilance and orientation, lateral tongue bite, and involuntary loss of urine. After being found unconscious in his car, he regained consciousness during transport, despite not being fully oriented. In the hospital, the initial symptoms were interpreted as an early seizure of unknown origin. Cranial computed tomography (CT) with CT angiography (CTA) and CT perfusion (CTP) showed no sign of brain infarction nor an occlusion of the intracranial arteries. There was also no evidence of intracranial hemorrhage. The only conspicuity was an area of increased perfusion in the CTP assumed to by hyperperfusion following a seizure, the presumed clinical diagnosis supported by an elevated prolactin (28 µg/l, normal to 19 µg/l). During the first 24 h in hospital, the patient regained orientation and additionally reported reading problems the morning before admission. He also reported severe lumbar pain. The subsequent CT of the lumbar spine showed a fracture of the fourth lumbar vertebra, which was treated conservatively. Electroencephalography (EEG) showed no abnormalities, so that an MR imaging was requested to clarify the cause of the clinically assumed seizure. The first attempt had to be aborted due to severe back pain and shortness of breath, the latter caused by an undiagnosed and untreated constructive obstructive pulmonary disease. Only one FLAIR-sequence could be acquired, that showed a T2-hyperintense area of the left occipital lobe (the area with increased perfusion in the CTP). The second attempt was performed the following day with sufficient oxygen supply, intravenous pain medication, and monitoring. The initial FLAIR-sequence already showed a volume increase of the T2-hyperintense signal alterations, compared to the day before. In addition, there were several new hemorrhages within the altered brain parenchyma. The high-resolution T2-weighed sequences showed multiple flow voids as an indication of vessels, but no thrombosis of the large sinus or deep cerebral veins. After application of contrast agent, the suspected area showed an inhomogeneous contrast enhancement, especially above the tentorium. The initially performed CTA showed several small, tortuous vessels within the area with increased perfusion in reanalysis. The follow-up MRI showed a significantly increased edema within 1 day, an indicator for a higher bleeding risk (Fig. 1). Therefore, a diagnostic angiography was performed which revealed an arteriovenous fistula of the middle meningeal artery, draining in cortical veins, corresponding to Cognard III classification with an annual hemorrhage risk of 40% [2]. The fistula was consequently embolized with histoacryl and lipiodol because of the high bleeding risk (Fig. 2). After the intervention, the patient was monitored in the intensive care unit overnight and then transferred to a neurological ward. He showed no neurological symptoms and recovered completely from his temporary visual impairment.

## Discussion

Arteriovenous fistulas are an important differential diagnosis in between the group of intracranial arteriovenous shunts because of their risk of intracerebral hemorrhage. They represent a small proportion in this group, accounting for 10–15% of all intracranial arteriovenous malformations. They are considered as acquired lesions, although the exact etiology remains unclear. The most common way to explain the origin of arteriovenous fistulas is based on a thrombosis of the big cerebral sinus. The subsequent increase in venous blood pressure and the angiogenic factors released seem to promote the development of arteriovenous fistulas [3, 4].

There are several classifications for the pattern of venous drainage. A common one is the classification of Cognard in five stages, depending on the venous drainage in a sinus (I°) to pial veins (III°) up to spinal, perimedullar veins (V°) [5].

The risk of hemorrhage does not only depend on the higher stage, but also on the localization and the anatomy of venous drainage. Especially stenosis in draining veins of the fistula, no alternative draining pathways for the brain tissue, and contrast stagnation after DSA represent statistically significant risk factors for a severe outcome with hemorrhage or neurological manifestations [1].

In our case, the patient presented with a seizure as initial manifestation of his fistula. The first CT did not show hemorrhage nor definable edema. Only the follow-up MRI showed significant edema and a rapid increase of the edema within 1 day, which was located exclusively within the white matter without involvement of the cortex. This made ischemia as primary cause of the described changes unlikely. The follow-up MR scan, together with the CTA and the clinical course, allowed to make a suspected diagnosis, which was confirmed by DSA followed by embolization.

The conclusions and main findings of this case are the following:

1. Frequent symptoms in arteriovenous fistulas are (depending on their localization) seizures and pulsatile tinnitus. The occurrence of seizures requires immediate (MR-) imaging to identify a fistula as possible cause due to their risk of hemorrhage.
2. The rapid increase of the edema and the fact, that it did not reach the cortex, suggested a different cause than ischemia for the MR T2-signal alterations. The first CT did not show any white matter hypodensity as a sign of edema.
3. Retrospectively, the increased perfusion in the initial CT and the torqued vessels in the CTA matched the changes seen in the MRI.
